# Neovascularization in Human Intracranial Atherosclerotic In-Stent Restenosis

**DOI:** 10.3390/diagnostics11020322

**Published:** 2021-02-17

**Authors:** Yiding Feng, Adam A. Dmytriw, Bin Yang, Liqun Jiao

**Affiliations:** 1 Department of Neurosurgery, Xuanwu Hospital, Capital Medical University, Beijing 100053, China; 3100102434@zju.edu.cn (Y.F.); yangbin_81@163.com (B.Y.); 2 Department of Interventional Neuroradiology, Xuanwu Hospital, Capital Medical University, Beijing 100053, China; 3 Neuroradiology & Neurointervention Service, Brigham and Women’s Hospital, Boston, MA 02215, USA; admytriw@bwh.harvard.edu

**Keywords:** intracranial atherosclerotic stenosis, vertebral artery, optical coherence tomography, neovascularization

## Abstract

Optical coherence tomography (OCT) has seen widespread use in cardiovascular and interventional endovascular imaging. While scattered reports of intracranial usage have been reported for the assessment of atherosclerotic stenosis, nutrifying neovasculature supplying plaque and neointima have not been demonstrated until now. We report the first in-vivo illustration of this phenomenon, which is a high-resolution depiction of a critical pathway for in-stent restenosis.

**Figure 1:**
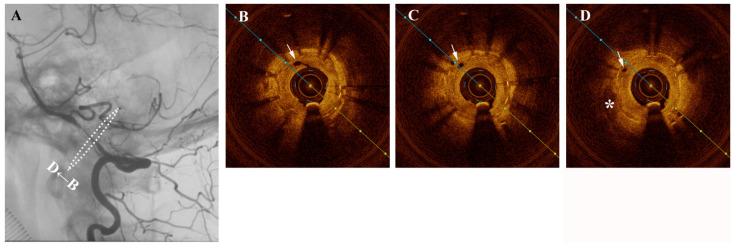
A middle-aged female presented one year after intracranial stenting for severe left intradural segment vertebrobasilar stenosis with recurrent vertigo. Digital subtraction angiography (DSA) confirmed severe in-stent restenosis and same-session optical coherence tomography (OCT) with an Ilumien Optis probe (St. Jude Medical, St. Paul, MN, USA) showed neovascularization-related neointimal hyperplasia (Figure 1). Figure 1 (**A**) Angiography confirming severe intracranial in-stent restenosis of the left intradural vertebral artery segment. Figure 1 (**B**–**D**) Pre-intervention cross-sectional optical coherence tomography (OCT) shows hypertrophic intima and hyporeflective microchannels (white arrow) originating directly from the lumen and penetrating contiguously towards the plaque (*). These are seen as hyporeflectile microchannels originating directly from the lumen, which penetrate contiguously towards irregular plaque and hyperplastic intimomedial complex. Neovascularization plays a role in intracranial atherosclerosis by providing trophic factors to plaque [1]. Such neovasculature is susceptible to intra-plaque hemorrhage and also importantly is a critical factor in the propagation of intimal hyperplasia following stenting [2]. Intracranial neovasculature cannot be resolved in-vivo by traditional imaging methods. In 2011, Mathews et al. performed OCT in the cavernous and the petrous segments of internal carotid artery, which used time-domain OCT assembled in a laboratory rather than a commercial product [3]. Although a small number of cases were enrolled, the result suggested that OCT examination of intracranial arteries was feasible. Until now, there have been numerous preliminary attempts at intracranial OCT use, but visualization of intracranial neovasculature has heretofore not been reported in a living patient. With the approximately 40 micron resolution afforded by OCT, an extremely rare and vivid look at the mechanisms behind plaque nutrition and neointimal hyperplasia of the intracranial vertebral artery is possible [4]. The in-stent stenosis was treated a Sequent Neo paclitaxel-coated balloon (B.Braun, Melsungen, Germany) for intimal hyperplasia inhibition (Figure 2). As plaque and neointimal morphologic characteristics are increasingly recognized as important contributors to disease prognosis and natural history, OCT may stand to inform treatment decisions and assess intimal tearing in the future.

**Figure 2:**
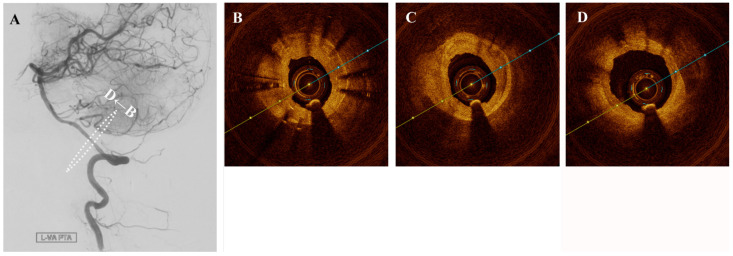
(**A**) Angioplasty with a drug-coated balloon was performed to recanalize successfully without significant intimal tear (**B**–**D**).

## Data Availability

Data are available upon reasonable request.
